# Association between inflammatory bowel disease and ankylosing spondylitis: Mendelian randomization of a different database and meta-analysis

**DOI:** 10.1097/MD.0000000000050015

**Published:** 2026-07-31

**Authors:** Jiawen Guo, Yingjie Qiao, Huadong Li

**Affiliations:** a Shandong University of Traditional Chinese Medicine, Jinan, Shandong, China; b Department of Tuina, Shandong University of Traditional Chinese Medicine Affiliated Hospital, Jinan, Shandong, China.

**Keywords:** ankylosing spondylitis, Crohn disease, inflammatory bowel disease, Mendelian randomization, ulcerative colitis

## Abstract

Inflammatory bowel disease (IBD) and ankylosing spondylitis (AS) are common autoimmune disorders that have a serious impact on the physical and mental health of patients. Some patients with AS also suffer from IBD, and some studies have shown that there is a link between the 2. However, the causal relationship between the 2 is not clear. This study aimed to infer a causal relationship between IBD and AS using Mendelian randomization (MR) analysis. We obtained genome-wide association study data related to IBD and AS from the Integrative Epidemiology Unit database and the Finnish database to infer the causal relationship between IBD and AS. MR analyses were performed using 3 methods, mainly inverse-variance weighting (IVW). The robustness of causal effects was ensured by multiple methods (instrumental variables were assessed using *F*-values, heterogeneity was detected by Cochran’s *Q*, horizontal pleiotropy was assessed by MR-Egger regression, and outliers were detected by Mendelian Randomization Pleiotropy RESidual Sum and Outlier and leave-one-out methods). Subsequently, we selected genome-wide association studies data from 3 different databases for an independent two-sample MR analysis and a meta-analysis based on 3 independent MR estimates to assess the causal relationship between IBD and AS. There was a positive causal relationship between Crohn disease (CD) and AS with IVW results (odds ratio = 1.19, 95% confidence interval = 1.12–1.27; *P* <.01). There was a positive causal relationship between ulcerative colitis (UC) and AS with IVW results (odds ratio = 1.60, 95% confidence interval = 1.28–2.00; *P* <.01). Neither CD nor UC had an inverse causal relationship with AS. In order to verify the accuracy of the results, we performed a meta-analysis of the results obtained from the 3 MR analyses, and the results were consistent with the results we had previously obtained. CD and UC may increase the risk of AS.

## 1. Introduction

Inflammatory bowel disease (IBD) is a group of chronic, recurrent, and noninfectious inflammatory diseases that commonly involve the gastrointestinal tract, including ulcerative colitis (UC) and Crohn disease (CD). Currently, there are millions of people suffering from IBD worldwide, and the incidence is continuously on the rise.^[1,2]^ IBD not only affects the human digestive system to some extent but also causes extraintestinal or systemic inflammation, such as fever, malnutrition, mouth ulcers, iritis, and may even lead to disability.^[3]^ Although the exact etiology of IBD remains unclear, it is widely believed to be caused by a variety of factors, such as genetic susceptibility, disturbances in the intestinal flora, dysregulated immune response, and environmental factors.^[4]^ Ankylosing spondylitis (AS) is an autoimmune disease characterized by inflammatory low back pain, morning stiffness, and limitation of spinal motion. It severely affects the function of the spinal joints, sacroiliac joints, and the tissues attached to the joints. The causes of AS are complex, involving genetic, environmental, and infectious factors. The disease is most common in young adults, with a prevalence rate of 0.1% to 0.6%, which seriously affects the physical and mental health of the patients and imposes a serious economic burden on society.^[5]^ Many patients with AS are clinically comorbid with IBD, and some studies have shown that IBD can induce systemic inflammation, which suggests that there is a correlation between the 2. However, there is no definitive conclusion at the genetic level as to whether there is a causal relationship between IBD and AS.

Randomized controlled trials are difficult to achieve due to various practical constraints, and traditional observational studies are susceptible to confounding factors and have limitations and unreliability in inferring causality. Mendelian randomization (MR) is a data analysis method for assessing etiological inferences in epidemiological studies, which uses genetic variants with strong correlations with exposure factors as instrumental variables to assess causal relationships between exposure factors and outcomes and is considered a natural randomized controlled trial.^[6]^ Because genetic variants are present at birth and remain stable throughout the life cycle, results derived from MR analyses can avoid confounders and reverse causality to a greater extent.^[7–9]^ Based on this, a two-sample MR test was designed to investigate the causal relationship between IBD and AS to provide some basis for the pathogenesis and treatment of both.

## 2. Materials and methods

### 2.1. Research design

In this study, single nucleotide polymorphisms (SNPs) significantly associated with exposure were selected as instrumental variables for the causal relationship between IBD and AS analyzed using a two-sample MR method. To ensure the reliability of the experimental results, the MR analysis needed to fulfill the following three key assumptions ^[10]^: SNPs in the final set of included instrumental variables were strongly associated with the exposure (IBD or AS), SNPs were strongly associated with the known confounders (factors influencing the IBD or AS) independently of each other, and SNPs influenced the outcome (IBD or AS) only through exposure (IBD or AS). This two-way MR analysis was performed in 2 steps: traits associated with CD and UC were examined as exposures, while AS was examined as an outcome in the first step and vice versa in the second step.

### 2.2. Data sources

The CD and UC datasets were both obtained from the Integrative Epidemiology Unit database. The data for UC came from a genetics study of age-related diseases with a sample size of 417,932 and SNPs of 24,187,301^[11]^; the data for CD came from a European genome-wide association study with a sample size of 28,072 and SNPs of 94,57,998.^[12]^

Genetic data on AS were obtained from the FinnGen Biobank, which collects and analyzes genomic and health data from 500,000 Finnish participants, with a sample size of 164,682 and several SNPs of 16,380,022. Table 1 shows the data involved in the study.

**Table 1:** Basic information of the GWAS database in the two-sample Mendelian randomization study.

Name of the disease		Database ID		Sample size		Race (of people)		Database	Sex		Vintages	
Crohn disease	ebi-a-gcst004132		28,072		European		The IEU GWAS Database			Hybrid		2017
Ulcerative colitis	ebi-a-gcst90018933		417,932		European		The IEU GWAS Database			Hybrid		2021
Ankylosing spondylitis	finn-b-M13_ANKYLOSPON		164,682		European		The FinnGen Biobank			Hybrid		2021

IEU = Integrative Epidemiology Unit, GWAS = genome-wide association studies.

Exposures and outcomes from different databases avoid the possibility of sample overlap, and the higher the sample overlap, the greater the bias it produces. The summarized data used in the article were obtained from public data and are freely available for download. Each genome-wide association studies (GWAS) covered in the article received ethical approval from its respective institution.

### 2.3. Selection of instrumental variables

The thousand base pairs project was used as the reference for this study,^[13]^ and SNPs that were significantly correlated with the exposure factors and independent of each other were selected, and the correlation parameters were set as *P* <5 × 10^-8^, *F* > 10 for a single SNP, calculated by the formula (*R*^2^ = 2 × (1−minor allele frequency) × minor allele frequency × β^2^, *F* = *R^2^* × (n−k−1)/[k × (1−*R^2^*)]). To exclude the interference of chained imbalance, the threshold of the chained imbalance parameter *R^2^* was set to 0.001, kb = 10000.

The Mendelian Randomization Pleiotropy RESidual Sum and Outlier (MR-PRESSO) test was used to eliminate the SNPs with significant heterogeneity, and the valid SNPs that were significantly associated with the exposure factors were selected as the instrumental variables in this study. After the above screening, we ensured that the SNPs met the hypothesis of the MR analysis (hypothesis 1: the genetic instrumental variable must be significantly associated with the exposure). The resulting data were harmonized to remove unmatched data and palindromic sequences. We searched all phenotypes associated with the ending through published studies and the PhenoScanner V2 database (http://www.phenoscanner.medschl.cam.ac.uk) and excluded SNPs associated with confounders and outcomes to fulfill the MR analysis hypotheses (hypothesis 2: the genetic instrument must be independent of all confounders of the exposure-outcome relationship; hypothesis 3: the genetic instrument can only affect the outcome through the exposure and not via any alternative biological pathway [no horizontal pleiotropy]). The last remaining SNPs were used as instrumental variables in this study.

### 2.4. Statistical analysis

In this study, MR was performed using the “TwoSampleMR” package in R 4.2.0 (MRC Integrative Epidemiology Unit [IEU], University of Bristol), and the inverse-variance weighting (IVW) method was used as the primary method to assess the causal relationship between IBD and AS. In the absence of heterogeneity, a fixed-effects model was used. When heterogeneity was present, a random-effects model was used. The reliability of the IVW results was assessed by using the MR-Egger regression method and the weighted median method (WMM) for auxiliary causal inference, and the results of the MR analyses were considered to be reliable if the directions of the above results were consistent with those of the IVW method. The IVW method estimates the causal effects of genes on traits by weighting the causal effects of different genetic variants on traits and then combining the estimated effects after weighting. The advantages of the IVW method are to reduce the effect of sample size, improve estimation accuracy, reduce bias, etc, so the study used it as the main analysis method.^[14]^ The other 2 methods are used as a supplement to the IVW method to determine whether there is consistency in the results of the MR analysis through beta values to enhance the robustness of causality. The WMM mainly assigns different weights to different genetic variants to reduce the influence of extreme genetic variants on causal inference and improve the stability of the results. The MR-Egger method is also capable of detecting multiplicity (intercept *P* <.05) and adjusting for confounding bias to a certain extent, thereby improving the accuracy of causality estimation.^[15]^

### 2.5. Sensitivity analysis

To strengthen the robustness of the results, a series of tests were performed. The IVW method and the MR-Egger method were used for the heterogeneity test; when *P* >.05, it was considered that there was no heterogeneity among SNPs. Multiplicity was analyzed by the MR-PRESSO method; when *P* >.05, it indicated that there was no multiplicity. The presence of heterogeneity among the instrumental variables was evaluated by the *Q*-test (Cochran’s *Q*), and a *P*-value <.05 indicated the presence of heterogeneity. Sensitivity analysis was performed, and the results were visualized using the leave-one-out method, and single SNPs were excluded to observe whether there was any effect on the final results.

### 2.6. Meta-analysis

In order to verify the accuracy of the results, 3 MR analyses were performed, and the meta-analysis results generated by the IVW method in the 3 MR analyses were subsequently combined. In the test of combined effect sizes, *P* <.05 indicated statistical significance, and when *P* >.05, there was no statistical significance. The heterogeneity test was used to determine whether the incorporated analyzed databases were heterogeneous or not, and we considered that there was no consistency when *P* >.1, and heterogeneity was indicated when *P* <.1. The smaller the *I*^2^, the less heterogeneity it represented.

All MR tests were performed using the R packages “TwosampleMR” and “MR-PRESSO” in the R statistical software (version 4.1.2; R Foundation for Statistical Computing).

## 3. Results

Analysis causal effects between IBD and AS using different MR methods. Analyses were conducted using the conventional IVW, WMM, and MR-Egger methods.

### 3.1. MR analysis results

**Figure 1.**
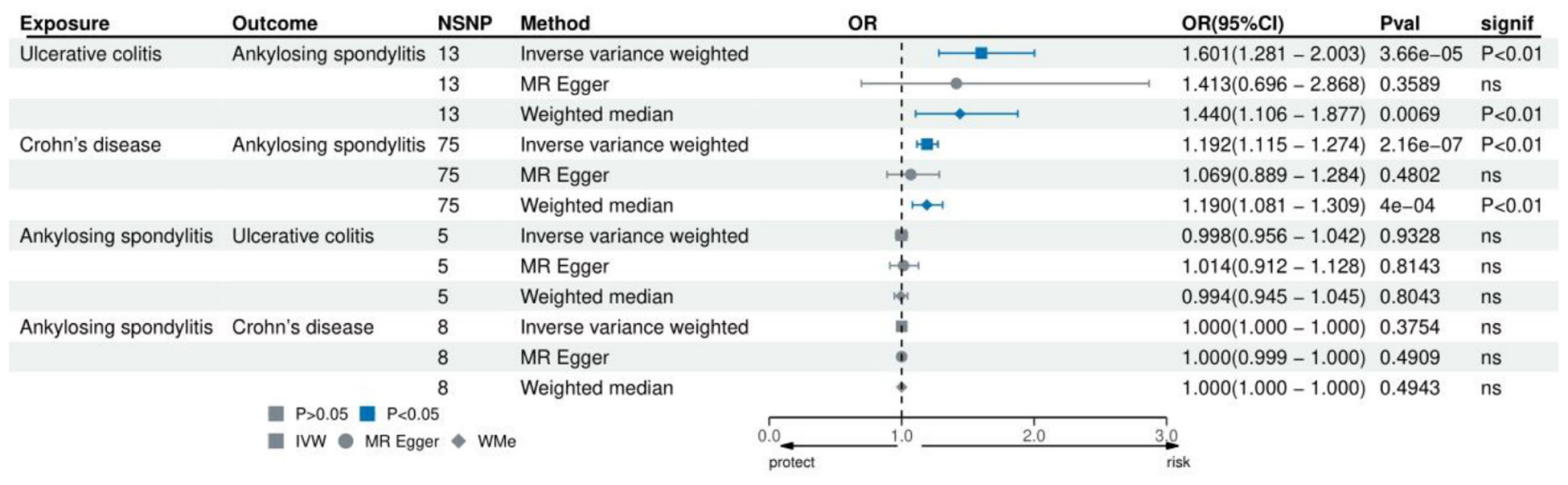
Mendelian randomization results. IVW = inverse-variance weighting, MR = Mendelian randomization, OR = odds ratio.

**Figure 2.**
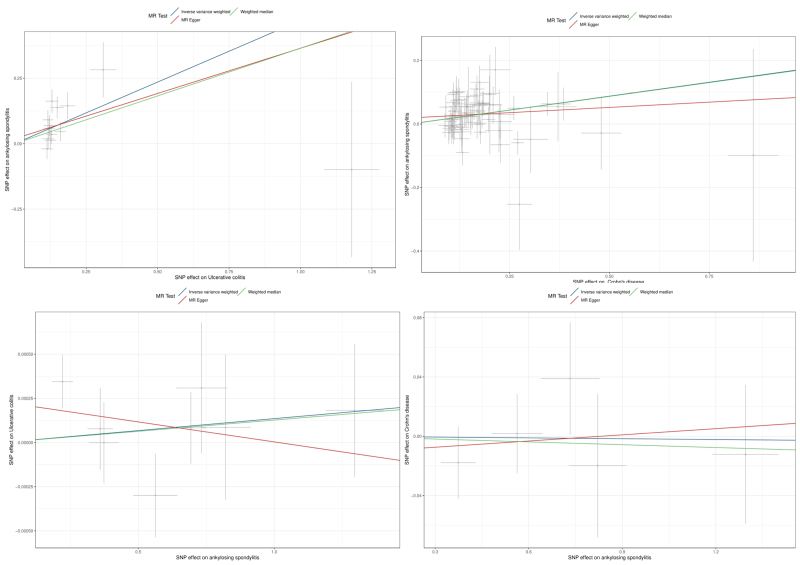
Scatter plot of Mendelian randomization analysis. MR = Mendelian randomization.

**Figure 3.**
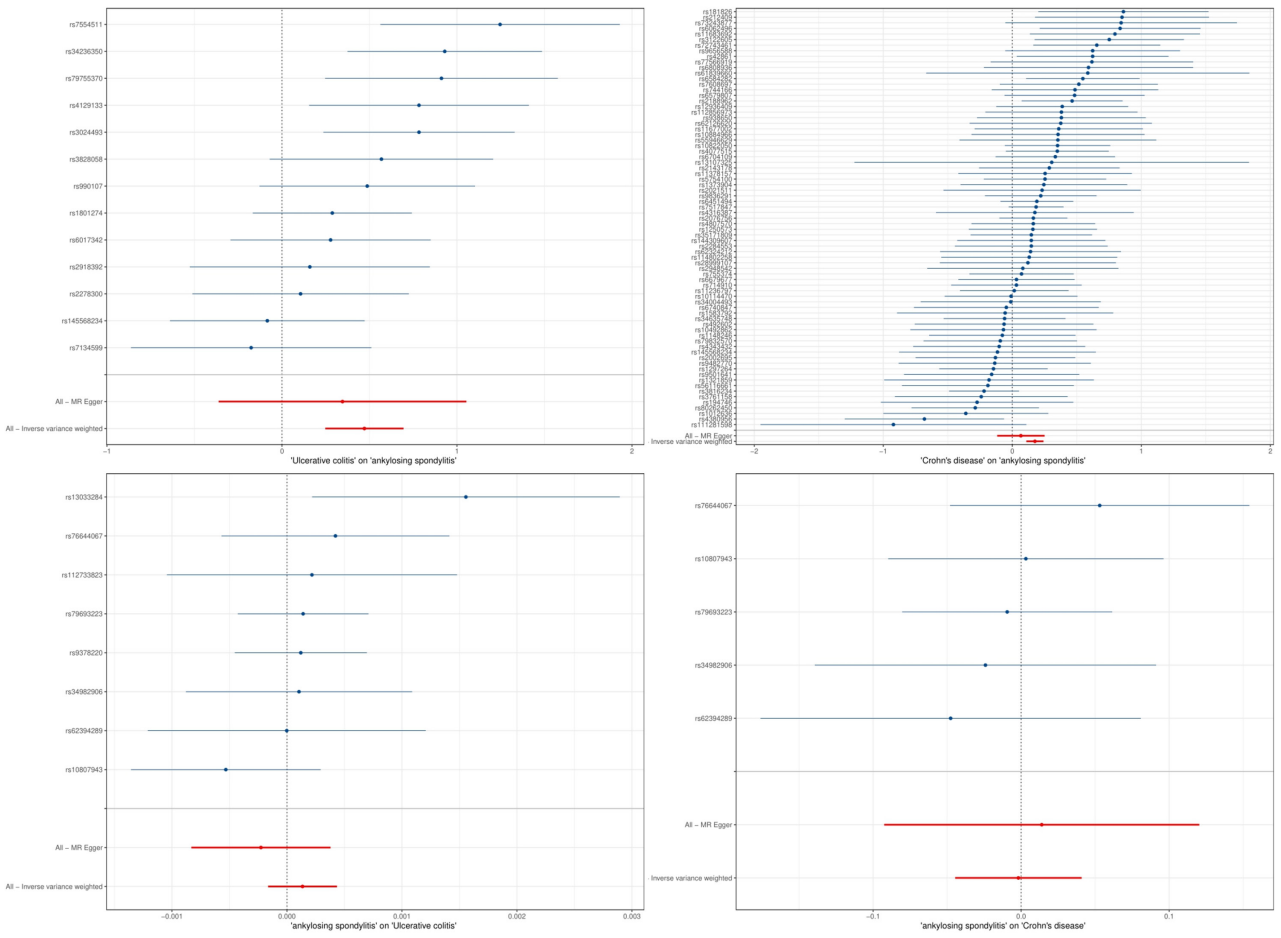
Forest plot for Mendelian randomization analysis. MR = Mendelian randomization.

Our MR analysis revealed a causal relationship between IBD and AS (Fig. 1). Analysis by the IVW methodology showed potential positive causality with CD and UC when AS was used as the dataset for the outcome CD versus AS (OR = 1.19, 95% confidence interval (CI) = 1.12–1.27; *P* <.01) and UC versus AS (OR = 1.60, 95% CI = 1.28–2.00; *P* <.01). There was no causal relationship with CD and UC when AS was used as the exposed dataset. Scatter plots and forest plots of MR analysis are shown in Figures 2 and 3.

### 3.2. Sensitivity analysis

**Figure 4.**
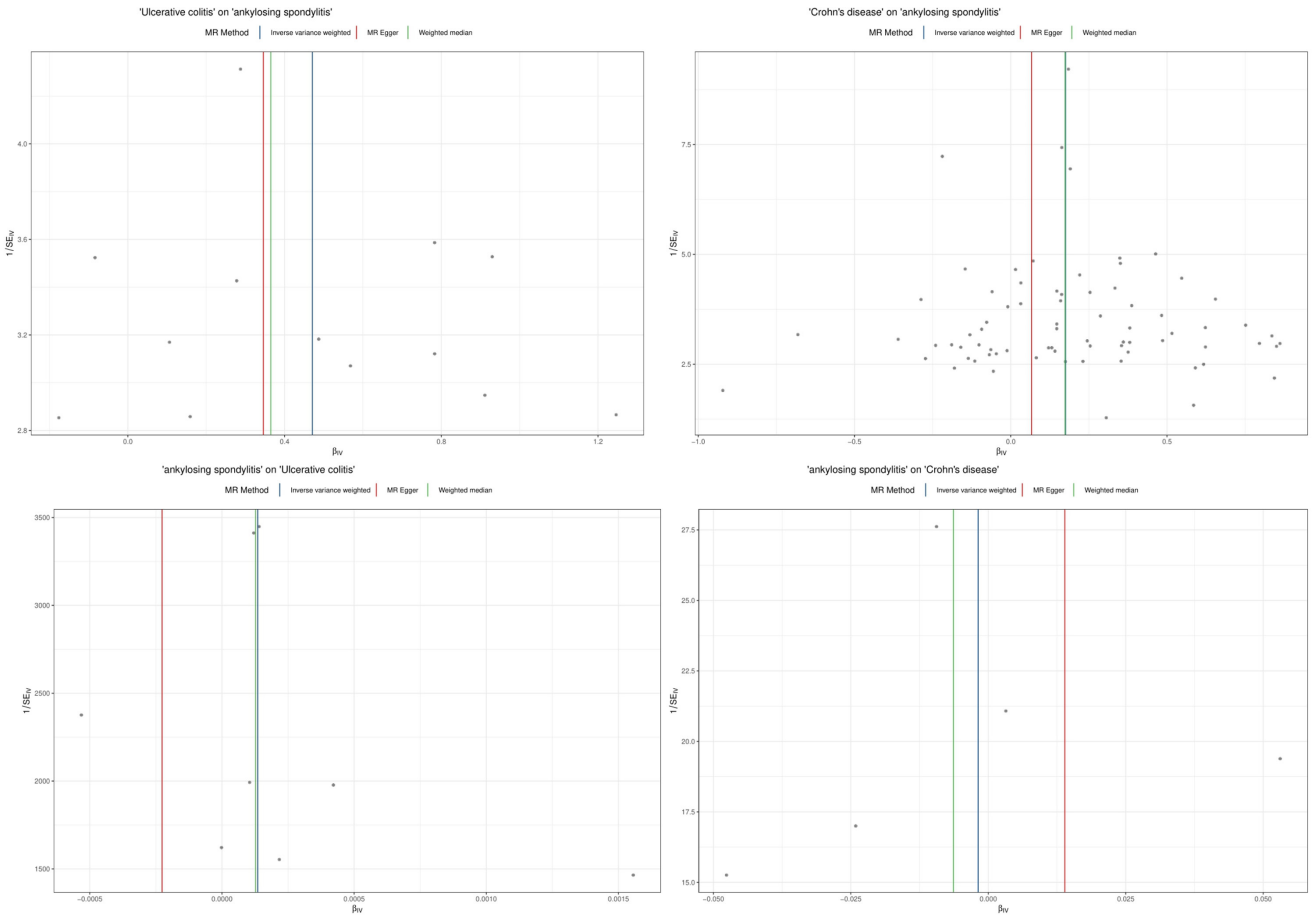
Funnel plot of Mendelian randomization results for 2 samples. MR = Mendelian randomization.

**Figure 5.**
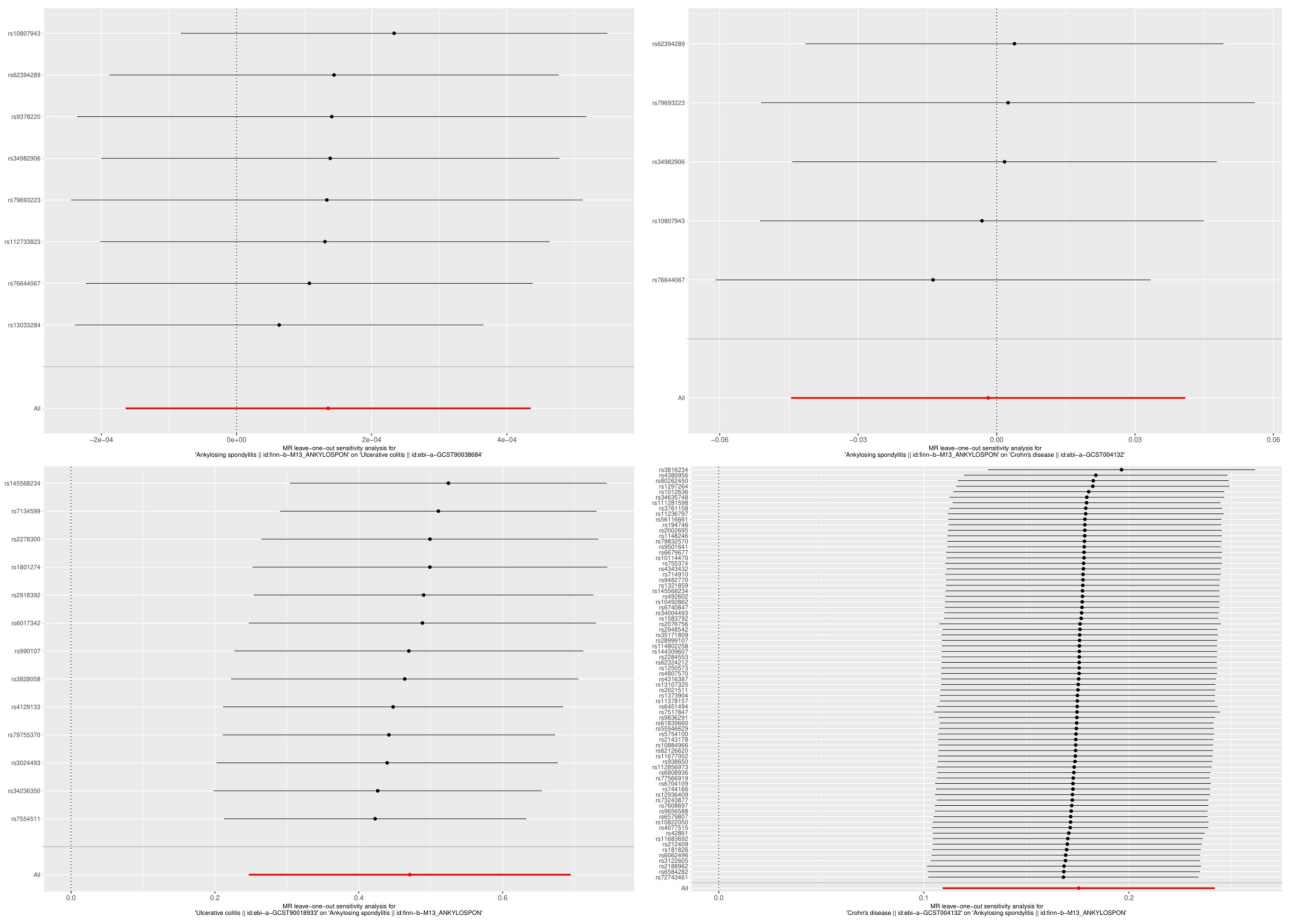
Results of leave-one-out sensitivity analysis. MR = Mendelian randomization.

The results of Cochran’s *Q* test showed that there was no heterogeneity (*P* >.05) in the SNPs obtained from our screening, so the fixed-effect model was used for the analysis. The MR-Egger intercept was utilized to test for horizontal pleiotropy, and the results showed that the *P* >.05 of its intercept proved that horizontal pleiotropy was not significant and that the results were more robust. To further test the stability of the above results, the distribution of causal effects shown in the plotted funnel plot was largely symmetrical and not biased by potential factors (Fig. 4). The leave-one-out test analysis showed that no individual single nucleotide polymorphisms had an impact on the overall causal estimates (Fig. 5).

### 3.3. Meta-analysis of MR results of databases from different sources

**Figure 6.**
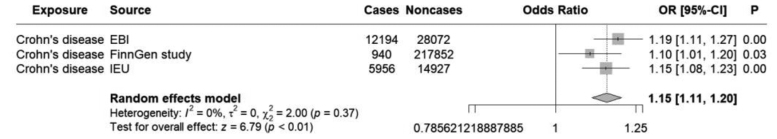
Meta-analysis of Crohn disease and ankylosing spondylitis. CI = confidence interval, EBI = European Bioinformatics Institute, IEU = Integrative Epidemiology Unit.

**Figure 7.**
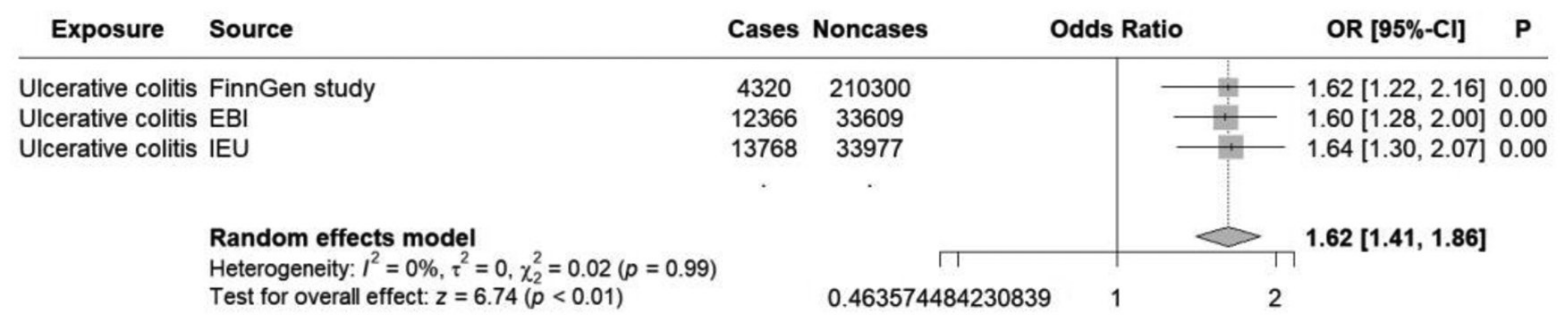
Meta-analysis of ulcerative colitis and ankylosing spondylitis. CI = confidence interval, EBI = European Bioinformatics Institute, IEU = Integrative Epidemiology Unit.

In order to verify the accuracy of our above findings, we selected 2 more databases from different sources for MR analysis, which were subsequently meta-analyzed with the above results, showing that UC was associated with AS (OR = 1.624, 95% CI = 1.41–1.86; *P* <.01) and CD with AS (OR = 1.15, 95% CI = 1.11–1.20; *P* <.01). The results of the heterogeneity test showed that there was no heterogeneity. This is consistent with the results of our previous study and increases the confidence of our study (Figs 6 and 7).

## 4. Discussion

UC is a class of chronic nonspecific inflammatory diseases, the pathogenesis of which is still unclear and may be mainly related to infection, immunity, and other factors. Some studies have shown that the susceptibility of different individuals to IBD depends largely on the interaction between the gut microbiota and the host immune response.^[16]^ In addition, 1 clinical study found a trend toward recovery of gut dysbiosis in patients treated with adalimumab.^[17]^ AS is a common, chronic, immune-mediated inflammatory disease with a complex pathogenesis that is thought to be the result of a combination of infectious, genetic, and environmental factors, although the exact pathogenesis has yet to be fully elucidated. An emerging body of science studying the microbiome has elucidated the important relationship between alterations in the gastrointestinal microbiota and autoimmune rheumatic diseases.^[18,19]^ Some bacteria play an important role in the pathogenesis of AS, such as *Klebsiella pneumoniae*^[20]^ and *Mycobacterium avium*.^[21]^ Among them, the association between AS and *K pneumoniae* has been intensively studied but with conflicting results. Initial studies showed that patients with AS had increased amount of this pathogen in their feces compared with the normal population.^[22]^ However, some researchers have also pointed out that there is no correlation between fecal carriage of *K pneumoniae* and disease activity. Most of the current studies maintain the same view as the former.^[23]^ Another study showed that compared with healthy individuals, AS patients had altered colony structure of *M anisopliae*, decreased diversity of the remaining intestinal flora, and increased expression of pathogenic bacteria, which correlated with AS disease activity, suggesting that the intestinal flora is involved in the onset and development of AS to some extent.^[24]^

Notably, immune modulation is also known to play an important role in the pathogenesis of AS. An imbalance of the intestinal flora can alter the intestinal barrier function or affect the intestinal flora and its metabolites, thus directly or indirectly altering the immune response of Th17 immune cells. The concept of the “gut-joint immune axis” has been proposed since then. The gastrointestinal tract of mammals is colonized by trillions of microorganisms, which are called intestinal microecology. In healthy individuals, this bidirectional interaction between the gut flora and the immune system is well balanced, but if this homeostasis is disrupted, the corresponding abnormality is known as intestinal dysbiosis, which can lead to digestive and nondigestive disorders such as IBD and metabolic disorders.

There is growing evidence that intestinal inflammation is associated with the dysbiosis that occurs in rheumatic diseases.^[25]^ Interactions between dysbiosis and the intestinal immune system can lead to abnormal activation of immune cells. Subclinical intestinal inflammation in patients with AS represents the extent to which immune cells are activated and correlates with the severity of spinal inflammation.^[26]^ There are common genetic risk factors between AS and IBD, and changes in the composition of the gut microbiota are observed in both diseases. In addition to this, there are numerous clinical cases of patients with both IBD and AS. This suggests that the 2 may be potentially linked or share a common pathogenesis, such as dysbiosis of the gut flora.^[16,27]^ The intestinal epithelium is an important physical and biochemical barrier against commensal and pathogenic microorganisms, protecting the host from microbial interactions and maintaining tissue homeostasis. Dysbiosis of the intestinal flora can lead to a compromised mucosal barrier and increased infiltration of commensal flora, which is important in the development and progression of disease. It has been found that intestinal permeability is increased in patients with AS and that lipopolysaccharide, a toxic component of endotoxin, enters the bloodstream and can induce a systemic inflammatory milieu by activating and inducing immune cells.^[28]^ Some preliminary evidence suggests that high serum levels of lipopolysaccharide and fatty acid–binding proteins may be present in AS, which is significantly associated with intestinal permeability. Many immune cells are present in the intestinal mucosa. Ecological dysregulation triggers these cells to increase pro-inflammatory cytokines and decrease anti-inflammatory cytokines, which activate the interleukin-23/interleukin-17 pathway and increase the T-helper 17 cells/regulatory T cells ratio, ultimately triggering or exacerbating inflammation in the gut and joints.

Numerous studies have shown that the 2 share common pathogenic factors and are somewhat correlated, but whether they are causally related remains to be further explored. This article is the first to use MR and rigorous genome-wide association analysis to further elucidate the causal relationship between IBD and AS. The results suggest that genetically predicted IBD is a risk factor for AS. The results of the analysis of IBD and AS were statistically significant by the IVW method and the WMM method, and the results of the rest of the methods were in the same direction. Therefore, this result suggests that the causal effect of IBD and AS remains stable. However, there was no MR evidence to support a potential reverse causality between IBD and the risk of AS. The authors suggest that the mechanism may be the presence of a large number of inflammatory factors in the digestive tract of patients with IBD, which can exacerbate the dysbiosis of the intestinal flora and increase the intestinal permeability of the patients, allowing the bacteria in the intestines to enter the bloodstream to activate and induce the immune cells to create a systemic inflammatory environment, which ultimately triggers the inflammation of joints. We explored the causal relationship between the 2 at the genetic level and excluded the shared genetic risk factors, namely, the interference from intestinal flora imbalance.

First, we believe our results are reliable because MR explores causal associations between exposures and outcomes through genetic data, free from causal inversion and confounders. Second, MR use genetic variation as an instrumental variable to mimic the design of a randomized controlled trial. It is intermediate between observational studies and intervention trials, providing information about public health interventions when randomized controlled trials may not be feasible. Finally, the large sample size and robustly correlated SNPs help detect causal effects with high precision. In order to strengthen the accuracy of our findings, we selected databases from different sources for validation, which provided strong evidence for our findings. However, there are some limitations to this study. Studies have shown that the prevalence of IBD in patients with AS in Western countries is 5% to 10%,^[29]^ while the incidence of IBD in the Han Chinese population with AS is only 0.4% to 0.6%.^[30]^ Although genome-wide association studies have confirmed the existence of common risk alleles between these 2 diseases, the above clinical observations suggest that ethnicity may be an important factor contributing to the discordant frequency of coexistence of AS and IBD in Caucasian and Han Chinese populations. Therefore, the data involved in this study are from European populations, which are not representative of the Chinese population or even the global population. On the other hand, these samples may also include a very small proportion of individuals from non-European populations, which may lead to bias in the instrumental variables for population stratification. Second, patients with AS have age and gender predispositions, but the database used in this study lacked detailed subgroup data to explore the effects of different genders or different ages on causal associations.

## 5. Conclusion

This article is a genetics-based causal study that explores the causal relationship between IBD and AS at the genetic level by using a two-sample MR analysis with publicly available databases and a large-scale GWAS study. The results show that there is positive causality between IBD and AS, which provides data support for both the basic research and the clinical field. However, there is still a lack of high-quality controlled studies, which may be an important entry point for future research. Therefore, researchers and clinicians must cooperate to increase the basic and clinical research efforts, exploring the in-depth IBD and AS episodes of the pathological mechanism, which has important clinical significance for the treatment of IBD and AS, rehabilitation, and complication prevention.

## Acknowledgments

We acknowledge all the genetics consortiums for making the GWAS summary data publicly available.

## Author contributions

**Conceptualization:** Jiawen Guo.

**Data curation:** Jiawen Guo.

**Formal analysis:** Yingjie Qiao.

**Investigation:** Yingjie Qiao.

**Methodology:** Yingjie Qiao.

**Funding acquisition:** Huadong Li, Yingjie Qiao.

**Writing – original draft:** Jiawen Guo.

**Writing – review & editing:** Huadong Li.
